# Case report: A novel case of paraneoplastic voltage gated calcium channel antibodies secondary to appendiceal adenocarcinoma

**DOI:** 10.3389/fneur.2024.1355437

**Published:** 2024-03-27

**Authors:** Ghanshyam Patel, Ahmet Sakiri, Abby Brown, Arfa Pasha, Vibhav Bansal

**Affiliations:** ^1^Mercyhealth, Internal Medicine Residency, Rockford, IL, United States; ^2^Department of Medicine, University of Illinois College of Medicine, Rockford, IL, United States; ^3^Department of Medicine, Sheikh Khalifa Medical City, Abu Dhabi, United Kingdom; ^4^Department of Neurology, Mercyhealth Javon Bea Hospital Rockford, Rockford, IL, United States

**Keywords:** VGCC, VGCC – voltage-gated calcium channels, paraneoplastic syndrome, appendiceal adenocarcinoma, carcinoma induced antibodies

## Abstract

Voltage gated calcium channels (VGCCs) play a critical role in neural transmission. Antibodies that target these ion channels can disrupt cellular signal transmission resulting in various clinical presentations. VGCC antibodies are most commonly associated with paraneoplastic syndromes such as Lambert-Eatons myasthenic syndrome. Here, we report a 47-year-old female with Stage IV appendiceal adenocarcinoma status post appendectomy and right hemicolectomy, who presented with progressive memory impairment, aphasia, ataxia, weakness, and headache. Neurologic exam was notable for right-sided parietal drift, decreased right arm swing, and ataxia of the bilateral upper extremities, more prominent on the right side. MRI of the brain with and without contrast was unremarkable. Cerebrospinal fluid (CSF) was notable for an elevated myelin basic protein (4.9 ng/mL, normal reference 0.0–3.7 ng/mL) with normal cell count, flow cytometry, and cytology. An extensive serum autoimmune neurology antibody evaluation revealed elevated VGCC autoantibodies (observed value: 96.1 pmol/L, normal range 0.0–30.0 pmol/L). A diagnosis of paraneoplastic voltage gated calcium channel antibodies secondary to appendiceal adenocarcinoma was made. The patient was treated with five exchanges with plasmapheresis over 10 days with significant clinical improvement in her symptoms. Upon literature review, this would be the first reported case of VGCC antibodies associated with appendiceal adenocarcinoma.

## Introduction

Voltage gated calcium channels play a critical role in cellular signaling and neural transmission. When activated, these ion channels trigger calcium influx into the cell, which triggers neurotransmitter release (1). This sequence can have a variety of effects on the post-synaptic cell, including muscle contraction in skeletal, cardiac, and smooth muscle cells. VGCCs also play a role in hormone secretion, gene expression, enzyme activation, and many other physiologic processes (1). Lambert-Eaton myasthenia syndrome (LEMS) is an autoimmune neuromuscular junction disorder where antibodies are produced against VGCCs, which can manifest as a paraneoplastic syndrome most commonly associated with small cell lung cancer (SCLC). We present a unique case of VGCC antibodies associated with appendiceal adenocarcinoma.

## Case report

**Figure 1:**
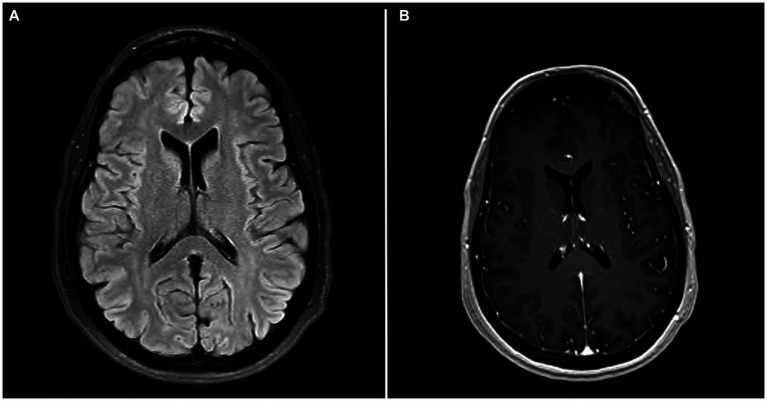
**(A)** MRI brain axial T2 FLAIR. **(A)** showing scattered minimal T2 and FLAIR hyperintensities in the periventricular and subcortical white matter. **(B)** MRI brain axial T1 with contrast showing no evidence of enhancement.

A 47-year-old female with a history of migraines initially presented with progressive right lower quadrant (RLQ) pain radiating to the periumbilical region that partially improved with antacids. CT scan showed perforated appendicitis with a large RLQ abscess (Figure 1). Patient was treated conservatively with antibiotics and IR guided drainage of the abscess. The patient then underwent a laparoscopic appendectomy with biopsy. The histopathological specimen revealed a positive *KRAS* mutation and well-differentiated grade I invasive appendiceal adenocarcinoma measuring 3.5 cm with invasion of the visceral peritoneum. Lab results were significant for a carcinoembryonic antigen (CEA) titer level of 9.0 ng/mL (normal reference 0.0–3.0 ng/mL). Further investigation with PET imaging showed residual adenocarcinoma in the terminal ileum, and the patient subsequently underwent a laparoscopic right hemicolectomy. Pathological analysis of 24 lymph nodes were negative for metastatic disease but invasion to the visceral peritoneum and abdominal wall was noted. The staging of the tumor was pT4a, pN0, pM1a (clinically Stage IV). Follow-up PET scan was negative for any local recurrence or distant metastases.

Two months after surgical intervention, the patient presented with fatigue, progressive memory impairment, concentration difficulties, issues with balance, weakness, and bilateral frontal headaches. Both short term and long-term memory was impaired, along with working memory, procedural memory and declarative memory. The patient was no longer able to perform routine tasks at work and instrumental activities of daily living such as grocery shopping were no longer possible. She had episodes of aphasia, which she described as difficulty getting words out that would last a few hours and spontaneously resolve. She had two falls recently, which she attributed to worsening imbalance, clumsiness, and weakness. She also reported daily tension-like headaches without pertinent associations.

**Figure 2:**
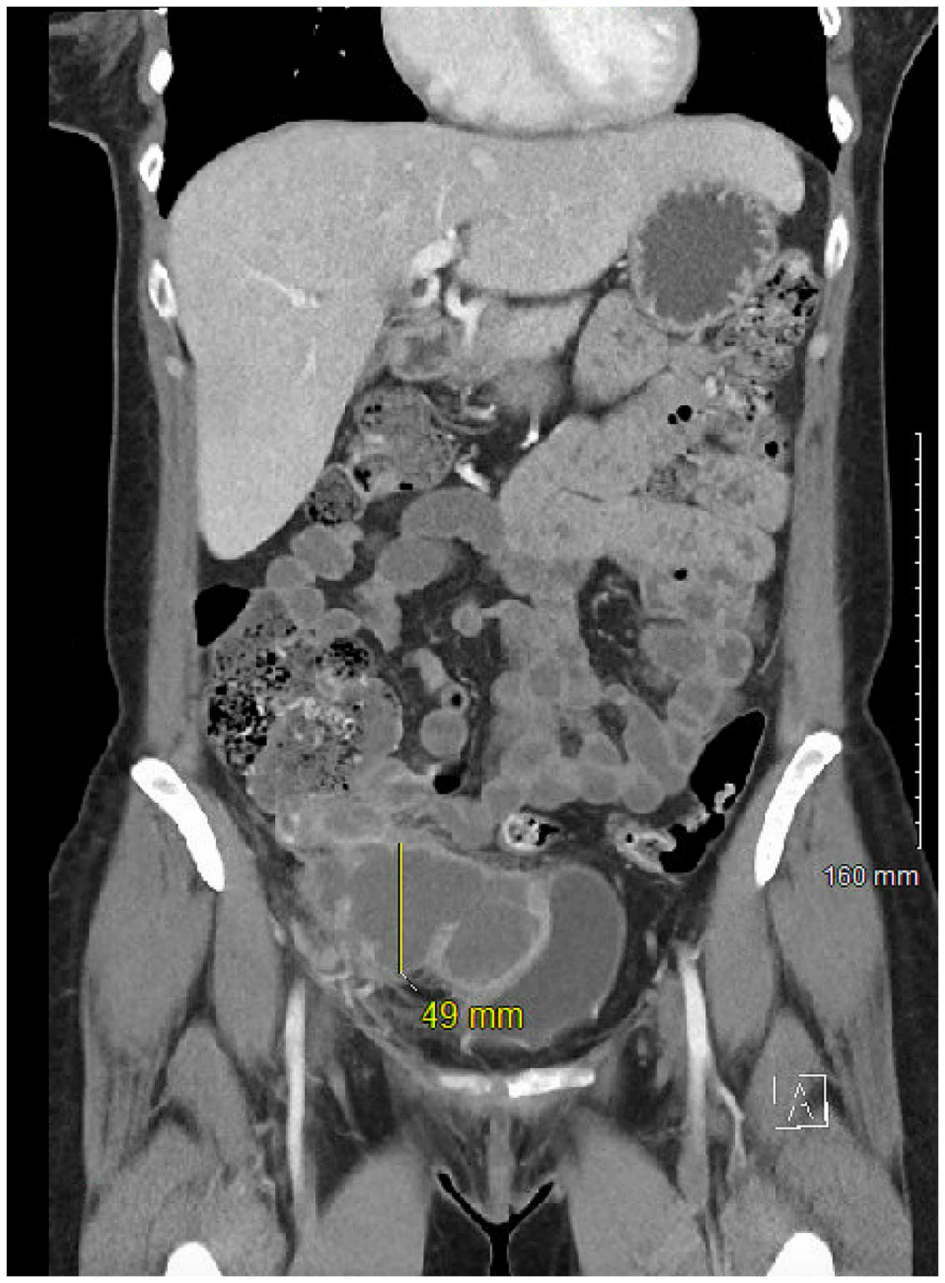
CT image of appendiceal adenocarcinoma.

Mental status exam was significant for poor concentration, inability to perform serial 7’s, one out of 3 memory recall after 5 min, and decreased processing speed. Neurologic exam was significant for parietal drift of the right arm, decreased right arm swing, and ataxia of the bilateral upper extremities, which was more pronounced on the right side. Additionally, the patient exhibited a broad-based gait. Bilateral biceps, triceps, brachioradialis, patellar, and Achilles reflexes were 2+ and symmetric. Romberg test was negative. No sensory deficits or other focal neurologic deficits were noted. MRI brain with and without IV contrast was unremarkable for evidence of brain metastasis; findings of scattered minimal T2 and FLAIR hyperintensities in the periventricular and subcortical white matter, presumed to be secondary to sequelae of headaches, were noted (Figure 2). The CSF analysis demonstrated normal glucose, cell count, and total protein. CSF findings (Table 1) were notable for rare B cells, presence of one oligoclonal band, and elevated myelin basic protein (4.9 ng/mL, normal reference 0.0–3.7 ng/mL). CSF flow cytometry and cytology studies were unremarkable. In view of recent diagnosis of appendiceal adenocarcinoma, paraneoplastic syndromes of the nervous system were considered. An autoimmune neurology antibody (Table 2) evaluation revealed elevated antibodies against VGCC (observed value: 96.1 pmol/L, normal range 0.0–30.0 pmol/L). VGCC antibodies secondary to paraneoplastic appendiceal adenocarcinoma was made. The antibody analysis was negative for Anti-HU, Anti-RI, Anti-YO, AMPA, NMDA, and GABA.

**Table 1:** Cerebral spinal fluid (CSF) characteristics.

Component	Reference range	Characteristics
Character CSF		Clear
Xanthochromia		Negative
Nucleated cells	<5/mm3	5
Glucose CSF	40–70 mg/dL	58
Total Protein CSF	15–45 mg/dL	33
Albumin CSF	8–37 mg/dL	20
Albumin serum	3.8–4.8 g/dL	4.4
IgG index CSF	0.0–0.7	0.6
CSF/serum albumin index	0–8	5
Immunoglobulin G QT serum	586–1,602 mg/dL	1,165
IgG CSF	0.0–6.7 mg/dL	3.1
IgG/Alb ratio CSF	0.00–0.25	0.16
IgG oligoclonal bands CSF		One (1) oligoclonal band

**Table 2:** Autoimmune neurology antibodies panel.

Component		Component	
Anti HU	Negative	Zic4 antibody	Negative
Anti RI	Negative	DNER antibody	Negative
Antineuruonal nuclear Ab Type 3	Negative	ITPR1 antibody	Negative
Anti YO	Negative	AMPA-R1 antibody	Negative
Purkinje Cell Cytop Ab Type 2	Negative	AMPA-R2 antibody	Negative
Purkinje Cell Cytop Ab Type Tr	Negative	GABA-B-R antibody	Negative
Amphiphysin antibody	Negative	NMDA-R antibody	Negative
CRMP-5 IgG S	Negative	GAD65 antibody	Negative
AGNA-1	Negative	Aquaporin 4 antibody	Negative
DPPX antibody	Negative	CASPR2 antibody, cell-based IFA	Negative
mGluR1 antibody	Negative	LGI1 antibody, cell-based IFA	Negative
IgLON5 antibody	Negative	VGCC antibody P/Q type (0.0–30.0 pmol/L)	96.1 (H)
Ma2/Ta antibody	Negative	Angio 1 Con Enz (14–82 U/L)	46

Patient was treated with plasma exchange (PLEX). A total of five exchanges (3 to 5 L of plasma each) over the next 10 days was performed. Within days of completing PLEX treatment, patient had significant improvement in her ataxia, weakness, and memory/concentration difficulties. Repeat neurologic exam showed full strength of all extremities, no signs of ataxia in the bilateral upper extremities, and normal gait. Patient was discharged home after completion of PLEX treatment. Patient was followed closely for 1 year with no recurrence of symptoms and repeat VGCC antibody testing was unremarkable (see Figures 1, 2).

## Discussion

VGCCs are located on the presynaptic terminal of the neural junction of many different types of cells, including skeletal muscle, cardiac muscle, smooth muscle, endocrine cells, and numerous others. The activation of VGCCs by action potentials allows calcium influx into the cell, which triggers neurotransmitter release and subsequent depolarization of the post synaptic membrane. This depolarization is responsible for many physiologic processes, including muscle contraction. VGCCs are divided into five types L, P/Q, N, R, T based on tissue and pharmacological properties (2) (see Tables 1, 2).

Antibodies against VGCCs are most commonly associated with LEMS but have also been linked to paraneoplastic cerebellar degeneration (PCD) and malignant hyperthermia. SCLC is the most common underlying malignancy noted in patients with VGCC antibodies. The rarely reported case suggests an association with non-SCLC, prostate cancer, and thymoma in adults (3–5). VGCC antibodies have also been reported in neuroblastoma and lymphoproliferative malignancy in children. Upon literature review, there has never been a reported case of VGCC antibodies associated with appendiceal adenocarcinoma. This would be the first noted case thus far.

The prevalence of VGCC antibodies in asymptomatic patients without known cancer is less than 2% (6, 7). Neoplasms of the appendix are extremely rare with an estimated rate of 0.12–2.6 cases per million people (8). There have been few isolated case reports of other paraneoplastic syndromes such as Myasthenia gravis and amphiphysin-IgG autoimmunity associated with appendiceal adenocarcinoma (8, 9). Most studies regarding LEMS as a paraneoplastic syndrome have been conducted in patients with SCLC. It has been shown that functional VGCCs are expressed on the surface membranes of cancer cells in SCLC which provides a probable mechanism for antibody development (10).

The management of paraneoplastic VGCC antibodies revolves around treatment of the underlying cancer. Stabilizing symptoms requires adequate tumor management with or without adjuvant therapy (11). In patients with malignancy-induced LEMS characterized by moderate–severe weakness that hinders daily function, the cornerstone of management is adequate immunomodulatory treatment along with treatment of the primary malignancy. Intravenous immunoglobulin therapy has been utilized in various cases of myasthenia gravis and has also been shown to have benefit in cases involving LEMS by reducing the number of VGCC antibodies in addition to clinical improvement of symptoms (12, 13). These studies suggest that long-term follow up with IVIG showed improvement in PEFR and electrophysiological measurements, and limb strength. These improvements were maintained at a 2 year follow-up period (14). The usual course of IVIG is a total dosage of 2 g/kg given over 2–5 days with maintenance therapy requiring repeat infusions at 4–12 week intervals showing satisfactory results. However, the peak effects of IVIG were seen to occur at 2–4 weeks, which is followed by a gradual return of muscle weakness (12, 15, 16).

Plasma exchange is another therapy for antibody-mediated disorders and has been utilized in several cases of LEMS (17–20). Typical protocol for plasmapheresis dictates 5 exchanges of approximately 3–5 L of plasma over the course of 1–2 weeks, similar to what was done for our patient. However, data suggests that the beneficial effects of plasmapheresis, which lead to clinical and electrophysiologic improvements, maybe short-lived. Trials involving combination of plasmapheresis with immunosuppressive therapy such as prednisone and azathioprine showed greater improvement than either regimen alone (20). Immunosuppressive therapy alone with agents such as prednisone, azathioprine, mycophenolate mofetil, cyclosporine, and even rituximab in refractory cases has been documented. However, the side effects associated with these agents require periodic surveillance and regular laboratory monitoring (21–23). Our patient did not have recurrence of symptoms during her 1 year follow up and repeat levels of VGCC antibodies were within the reference range. As with many paraneoplastic syndromes, the prognosis of these patients depends on the treatment and/or progression of the underlying neoplasm.

## Data availability statement

The original contributions presented in the study are included in the article/supplementary material, further inquiries can be directed to the corresponding author.

## Ethics statement

Written informed consent was obtained from the individual(s) for the publication of any potentially identifiable images or data included in this article.

## Author contributions

GP: Writing – original draft, Writing – review & editing. AS: Writing – original draft, Writing – review & editing. AB: Writing – original draft, Writing – review & editing. AP: Writing – original draft, Writing – review & editing. VB: Writing – original draft, Writing – review & editing.
